# IL-10 predicts incident neuroinflammatory disease and proviral load dynamics in a large Brazilian cohort of people living with human T-lymphotropic virus type 1

**DOI:** 10.3389/fimmu.2024.1416476

**Published:** 2024-06-19

**Authors:** Tatiane Assone, Soraya Maria Menezes, Fernanda de Toledo Gonçalves, Victor Angelo Folgosi, Marcos Braz, Jerusa Smid, Michel E. Haziot, Rosa M. N. Marcusso, Flávia E. Dahy, Augusto César Penalva de Oliveira, Evelien Vanderlinden, Sandra Claes, Dirk Daelemans, Jurgen Vercauteren, Dominique Schols, Jorge Casseb, Johan Van Weyenbergh

**Affiliations:** ^1^ Laboratory of Dermatology and Immunodeficiencies, Department of Dermatology, Medical School, University of São Paulo Brazil/Institute of Tropical Medicine of São Paulo, São Paulo, Brazil; ^2^ Laboratory of Immunohematology and Forensic Hematology-LIM40, Department of Forensic Medicine, Medical Ethics, Social Medicine and Work, University of São Paulo Medical School, São Paulo, Brazil; ^3^ Laboratory of Clinical and Epidemiological Virology, Department of Microbiology, Immunology and Transplantation, Rega Institute for Medical Research, KU Leuven, Leuven, Belgium; ^4^ Programa de Pós-graduação em Ciências da Saúde, Faculdade de Medicina da Bahia, Universidade Federal da Bahia, Salvador, Bahia, Brazil; ^5^ Institute of Infectious Diseases “Emilio Ribas” (IIER) de São Paulo, São Paulo, Brazil; ^6^ Laboratory of Virology and Chemotherapy, Department of Microbiology, Immunology and Transplantation, Rega Institute for Medical Research, KU Leuven, Leuven, Belgium

**Keywords:** HTLV-1, HAM incidence, cytokines, inflammation, biomarkers

## Abstract

Human T-Lymphotropic Virus type-1 (HTLV-1) is a unique retrovirus associated with both leukemogenesis and a specific neuroinflammatory condition known as HTLV-1-Associated Myelopathy (HAM). Currently, most proposed HAM biomarkers require invasive CSF sampling, which is not suitable for large cohorts or repeated prospective screening. To identify non-invasive biomarkers for incident HAM in a large Brazilian cohort of PLwHTLV-1 (n=615 with 6,673 person-years of clinical follow-up), we selected all plasma samples available at the time of entry in the cohort (between 1997–2019), in which up to 43 cytokines/chemokines and immune mediators were measured. Thus, we selected 110 People Living with HTLV-1 (PLwHTLV-1), of which 68 were neurologically asymptomatic (AS) at baseline and 42 HAM patients. Nine incident HAM cases were identified among 68 AS during follow-up. Using multivariate logistic regression, we found that lower IL-10, IL-4 and female sex were independent predictors of clinical progression to definite HAM (AUROC 0.91), and outperformed previously suggested biomarkers age, sex and proviral load (AUROC 0.77). Moreover, baseline IL-10 significantly predicted proviral load dynamics at follow-up in all PLwHTLV-1. In an exploratory analysis, we identified additional plasma biomarkers which were able to discriminate iHAM from either AS (IL6Rα, IL-27) or HAM (IL-29/IFN-λ1, Osteopontin, and TNFR2). In conclusion, female sex and low anti-inflammatory IL-10 and IL-4 are independent risk factors for incident HAM in PLwHTLV-1,while proviral load is not, in agreement with IL-10 being upstream of proviral load dynamics. Additional candidate biomarkers IL-29/IL-6R/TNFR2 represent plausible therapeutic targets for future clinical trials in HAM patients.

## Introduction

Human T-lymphotropic virus type-1 (HTLV-1) has been associated with both leukemogenesis and multiple inflammatory diseases (1). It is estimated that worldwide around 10 million people are living with HTLV-1 (further referred to as PLwHTLV-1), of which 1–5% will develop a specific neuroinflammatory condition called HTLV-1-Associated Myelopathy (HAM) (2). HAM is a debilitating and progressive disease that can severely impact a person’s quality of life (3). Because only a small fraction of those infected with HTLV-1 develop HAM, it is important to identify sensitive biomarkers that can be used to monitor PLwHTLV-1 (4, 5). Despite a partial overlap in proviral load (PVL) between asymptomatic PLwHTLV-1 and HAM patients, those with higher proviral loads are more likely to develop neurological symptoms and possible, probable or definite HAM as defined by the Castro-Costa criteria (6–8).

In addition to PVL, the expression and production of cytokines have been observed in patients with HAM in both peripheral blood and the central nervous system (CNS). A plethora of inflammatory cytokines (5) have been quantified in both cerebro spinal fluid (CSF), plasma/serum and cell culture supernatants of PLwHTLV-1 and HAM patients. Strikingly, almost all inflammatory cytokines are highly increased, even in asymptomatic PLwHTLV-1, due to virus-triggered polyclonal T-cell activation and proliferation. Although no single cytokine has been put forward as a definitive biomarker able to discriminate asymptomatic (ASY) from HAM, a major role for IFN-γ has been suggested (9). Downstream of IFN-γ, the chemokine CXCL10 has shown promise as a predictive biomarker for HAM clinical progression, but only when measured in CSF (10). On the other hand, anti-inflammatory cytokines IL-4, IL-10 and TGF-β have been detected in HAM. Of note, *in vitro* responsiveness to IL-10 and TGF-beta was decreased in HAM patients (10), while *in vitro* IL-10 production and downstream STAT3 signaling was found responsible for increased proliferation in HAM patient-derived T-cell clones (11).

Defining a set of biomarkers associated with incident HAM cases (iHAM) might help understand its neuroinflammatory pathogenesis but, more importantly, will help identify PLwHTLV-1 with the greatest disease risk for close monitoring and early therapeutic intervention. The objective of this study was to identify plasma inflammatory and pro-inflammatory cytokines as non-invasive and easy-to-use biomarkers able to predict iHAM in a large cohort, aiming towards affordable and robust large-scale testing to prevent pathogenesis in the millions of PLwHTLV-1 at risk of developing this untreatable neuroinflammatory disease.

## Methods

### Cohort characteristics, patient recruitment, sampling strategy and clinical evaluation

**Figure 1:**
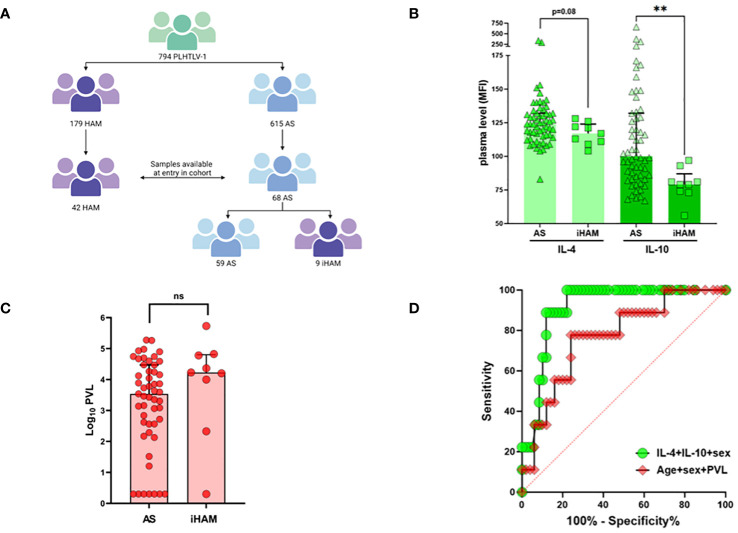
Low anti-inflammatory cytokines IL-4 and IL-10 predict incident disease (iHAM) in a large Brazilian cohort. **(A)** Flow diagram of the Sao Paulo cohort of People living with HTLV-1 and strategy for plasma sample selection. **(B)** Plasma levels of IL-4 and IL-10 in neurologically asymptomatic (AS) during follow-up and in individuals with a diagnosis of incident HAM (iHAM). **(C)** Proviral load levels in neurologically asymptomatic (AS) during follow-up and in individuals with a diagnosis of incident HAM (iHAM). **(D)** ROC curves distinguishing AS and iHAM based on age, sex and proviral load (red, AUC=0.78, p=0.010), or IL-4, IL-10 and sex (green, AUC=0.91, p<0.001).

We have previously studied HTLV-1 natural history in a large Brazilian cohort of PLwHTLV-1 (12, 13). Within this existing cohort, we performed a nested case-control study for incident HAM/TSP (cases), compared against matched controls who remained neurologically asymptomatic (AS) during the study period (August 1997 to December 2019). All First available plasma samples from PLwHTLV-1 were included, selecting those closest to the entry in the cohort Recruitment and sampling strategy are shown in **Figure 1A** and detailed in the Results section. All demographic, viral and immunological data for all PLwHTLV-1 included in this study are summarized in **Supplementary Table S1**. For HAM patients, plasma samples were obtained before standardized treatment (pulse therapy with methylprednisolone). Clinical evaluation and a standardized screening neurological examination were performed by a board-certified neurologist, with definite HAM defined by the criteria of Castro-Costa et al (7). Serological screening for HTLV-1 was performed at the “Emilio Ribas” Institute of Infectious Diseases, utilizing GOLD ELISA HTLV-1/2 (Diasorin, UK), followed by confirmation with Western Blot (MP Diagnostics, HTLV Blot 2.4^®^) and in-house nested PCR (13). Blood samples were collected in K3-EDTA (0.054 ml/tube), plasma was separated by centrifugation (15 min, 2500 rpm) and PBMC were purified by Ficoll density gradient centrifugation (GE Healthcare Life, USA). Cells were washed with saline solution, the cell number was adjusted to 10^6^ cells, followed by storage (as “dry pellet”) at -80°C. DNA was extracted utilizing a commercial kit (Illustra Tissue and Cells Genomic Prep Mini Spin kit, Fairfield, CA), according to the manufacturer’s instructions, and stored at -80°C. Data entry into the electronic database RedCap^®^ was carried out by two administrative assistants and verified by the first and last author for quality control.

### Quantif*i*cation of HTLV-1 proviral load

The amount of HTLV-1 provirus in samples was measured using real-time PCR, which specifically examined the virus’s pol gene using primers and probes, normalized against the human albumin reference gene. Samples were tested in duplicate to ensure precision, and results were reported as HTLV-1 DNA copies per 10^6^ PBMCs, and the minimum limit of detection was 10 copies per 10^6^ cells, with a theoretical maximum of 10^6^ per 10^6^ cells (100% infected cells) (14).

### Lymphoproliferation assay

Spontaneous T-cell proliferation assay was performed as described in detail elsewhere (15). Briefly, 10 mL of peripheral heparinized blood was collected from every patient and control, and PBMCs were isolated using Ficoll-Hypaque (Pharmacia, New Jersey, USA) gradient, washed two times in sterile saline and resuspended in RPMI 1640 (Difco, NY, USA). PBMCs from PLwHTLV-1 and controls (2 × 10^6^ cells/mL in RPMI with 10% fetal calf serum) were incubated at 37°C and 5% CO2 for three days for spontaneous proliferation (Costar, Cambridge, MA). Cells were pulsed with tritiated thymidine (0.5 μCi/mL, Amersham Int., England) 18 h before harvesting in a semi-automatic cell harvester (Flow Laboratories, United Kingdom), counted in a β-counter (Beckman, USA) and reported as mean counts per minute (CPM) of triplicate samples.

### Plasma cytokine levels

Plasma cytokines were measured using the CBA^®^ (Cytometric Bead Array, BD Biosciences) Human Th1/Th2/Th17 Cytokine Kit, including Interleukin-2 (IL-2), Interleukin-4 (IL-4), Interleukin-6 (IL-6), Interleukin-10 (IL-10), Tumor Necrosis Factor (TNF), Interferon-γ (IFN-γ), and Interleukin-17A (IL-17A) in all samples (n=110). For samples with sufficient volume, Luminex assays (CXCL10 and Bio-Plex Pro Human Inflammation Panel 1, 37-Plex, including APRIL/TNFSF13, BAFF/TNFSF13B, sCD30/TNFRSF8, sCD163, Chitinase-3-like 1/YKL-40, gp130/sIL-6Rβ, IFN-α2, IFN-β, IFN-γ, IL-2, sIL-6Rα, IL-8, IL-10, IL-11, IL-12 (p40), IL-12 (p70), IL-19, IL-20, IL-22, IL-26, IL-27 (p28), IL-28A/IFN-λ2, IL-29/IFN-λ1, IL-32, IL-34, IL-35, LIGHT/TNFSF14, MMP-1, MMP-2, MMP-3, Osteocalcin, Osteopontin, Pentraxin-3, sTNF-R1, sTNF-R2, TSLP, and TWEAK/TNFSF12) were performed (n=64, of which 37 AS, 6 iHAM and 21 HAM). Both immunoassays were performed in accordance with the respective manufacturers’ instructions.

### Ethical issues

The Ethical Board of “Instituto de Infectologia Emilio Ribas”, Sao Paulo-Brazil, approved the protocol (Number 07688818.2.1001.0061). Signed informed consent was obtained from all participants prior to study inclusion.

### Statistical analysis

We employed frequentist univariate and multivariate statistical analysis, including logistic regression, Mann-Whitney test, Spearman correlation, using XLStat, GraphPad Prism version 9, as well as machine learning algorithms from Weka (version 3.8.4).

## Results

### Low anti-inflammatory cytokines IL-4 and IL-10 and female sex predict incident neuroinflammatory disease in PLwHTLV-1

As shown in **Figure 1A**, the entire cohort consists of 794 PLwHTLV-1, of which 179 were diagnosed with definite HAM, and 615 were neurologically asymptomatic (AS) at entry in the cohort. During 6673 person-years of clinical follow-up (median 11 years, range 2–21), eleven incident cases of clinically definite HAM (iHAM) were observed among 615 AS (1.79%, 164.8/100,000 person-years) (**Figure 1A**). The vast majority of incident cases (9 out of 11, 81.8%) were women, consistent with a higher risk of clinical progression in female PLwHTLV-1^1^. For nine out of eleven incident cases (all women), a pre-diagnostic plasma sample was obtained, and compared to all available samples of PLwHTLV-1 who remained neurologically asymptomatic (AS) during follow-up (n=59). Lower systemic IL-10 and IL-4 levels (Mann-Whitney, respectively p=0.013 and p=0.08, **Figure 1B**) at entry in the cohort were observed in iHAM, as compared to AS. This was confirmed by univariate and multivariate logistic regression (**Table 1**), demonstrating that IL-10 [OR 0.93 (0.88–0.97)], IL-4 [OR 0.93 (0.89–0.99)], and sex [OR 0.031 (0.001–0.868)], were independent predictors of incident HAM, together resulting in excellent discriminatory power. However, age or proviral load were not significant predictors of incident HAM in either univariate or multivariate analysis (**Figure 1C**, **Table 1**). Thus, the inclusion of plasma cytokines improved iHAM classification (as evident from the ROC curve ROC AUC 0.91, p<0.0001, **Figure 1D**), as compared to previously identified risk factors age, sex and proviral load (ROC AUC 0.78, **Figure 1D**).

**Table 1:** Logistic regression analysis of iHAM risk factors.

Parameter	Univariate models			Multivariable Model 1			Multivariable Model 2		
	Odds ratio	95% CI	p-val	Odds ratio	95% CI	p-val	Odds ratio	95% CI	p-val
**Age**	1.002	0.968–1.036	0.920	1.003	0.964–1.044	0.889			
**Sex (male)**	**0.028**	**0.001–0.554**	**0.019**	**0.036**	**0.002–0.741**	**0.031**	**0.031**	**0.001–0.868**	**0.041**
**DNA HTLV-1 Proviral Load**	1.312	0.915–1.882	0.139	1.328	0.914–1.928	0.136			
**IL-2**	0.969	0.925–1.015	0.182						
**IL-4**	**0.940**	**0.893–0.989**	**0.017**				**0.925**	**0.866–0.987**	**0.019**
**IL-6**	0.999	0.996–1.002	0.420						
**IL-10**	**0.923**	**0.880–0.969**	**0.001**				**0.926**	**0.880–0.974**	**0.003**
**TNF**	0.964	0.924–1.007	0.097						
**IFN-γ**	0.992	0.970–1.015	0.492						
**IL-17A**	1.007	0.991–1.023	0.396						

Significant results are in bold.

### Plasma IL-10 predicts proviral load dynamics on follow-up across clinical groups

**Figure 2:**
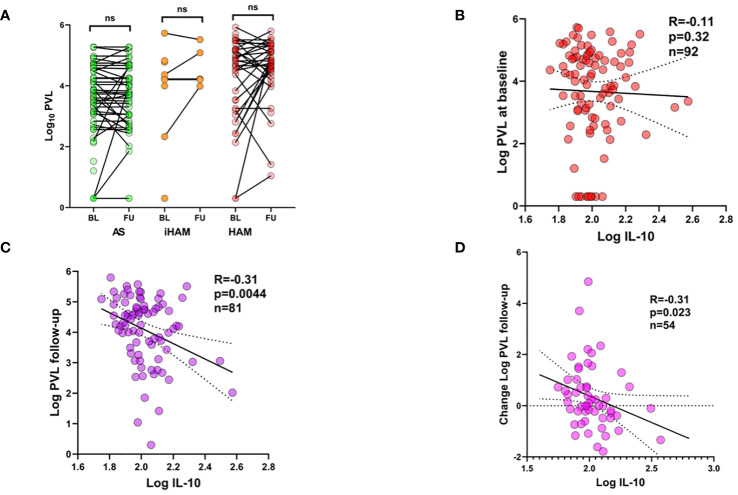
Plasma IL-10 predicts proviral load dynamics during follow-up of PLwHTLV-1. **(A)** Before-after plots of PVL at baseline (BL, entry in the cohort) and at first follow-up (FU). **(B)** Spearman correlation between plasma IL-10 at baseline and PVL at baseline **(B)**, PVL at follow-up **(C)**, and Change in PVL i.e. value follow-up minus baseline **(D)**.

Due to the unexpected lack of predictive power of PVL in iHAM in either univariate or multivariate analysis, we further investigated the possible temporal associations between plasma IL-10 and proviral load, both at sampling and during follow-up. **Figure 2A** shows the highly variable proviral load dynamics from baseline (entry in the cohort) to the first follow-up sample in all clinical groups (AS, iHAM and HAM). Due to this high variability, no significant decrease or increase in PVL was observed in any subgroup. Similar to our previous findings in AS and HAM (13), plasma IL-10 obtained before clinical progression did not correlate to PVL at baseline (**Figure 2B**, Spearman’s R=-0.11, p=0.23, n=92) across the cohort, and neither in iHAM, nor in joint analysis of AS+iHAM or iHAM+HAM. However, plasma IL-10 was significantly and negatively correlated to PVL at follow-up (**Figure 2C**, Spearman R=-0.31, p=0.0044, n=81), i.e. lower IL-10 predicts a subsequent rise in PVL across all clinical groups. This phenomenon was even more pronounced in AS+iHAM (R=-0.43, p=0.003, n=46) but completely absent in HAM (R=0.011, p=0.95, n=36), despite a similar sample size. No firm conclusions can be drawn on IL-10/PVL dynamics in iHAM, due to the recent diagnosis and short follow-up (n=9 at sampling, n=6 at follow-up), although the effect size is similar to the entire cohort (R=-0.30, p=0.68). Thus, IL-10 reaches a nadir in iHAM but increases in HAM patients, possibly provoking a rise in PVL. Since increased PVL upon follow-up is strongly associated with baseline PVL, we corrected for this possible bias by calculating the change in log PVL (Delta PVL). Again, we found that plasma IL-10 was also able to significantly predict Delta PVL (**Figure 2D**, R=-0.31, p=0.023, n=54).

IL-10 can act as a switch for lymphoproliferation in HTLV-1-infected T-cell clones derived from HAM patients (11). Thus, we investigated the possible correlation of plasma IL-10 with lymphoproliferation, as well as CD4 and CD8 levels in AS, iHAM and HAM, which might underlie its predictive effect on PVL dynamics. However, plasma IL-10 was not significantly correlated to lymphoproliferation (measured as described in Methods), CD4 or CD8 levels (data not shown). Since CXCL10 has been proposed as a candidate biomarker for clinical progression in HAM, we also tested its possible predictive role in PVL dynamics. Plasma CXCL10 at baseline (simultaneous with IL-10) was not correlated to PVL but positively correlated to PVL at follow-up (data not shown). However, upon correcting for baseline PVL, either by linear regression or by calculating Delta PVL, CXCL10 was no longer able to predict PVL dynamics over time, in contrast to IL-10.

### Additional plasma biomarkers for incident HAM

**Figure 3:**
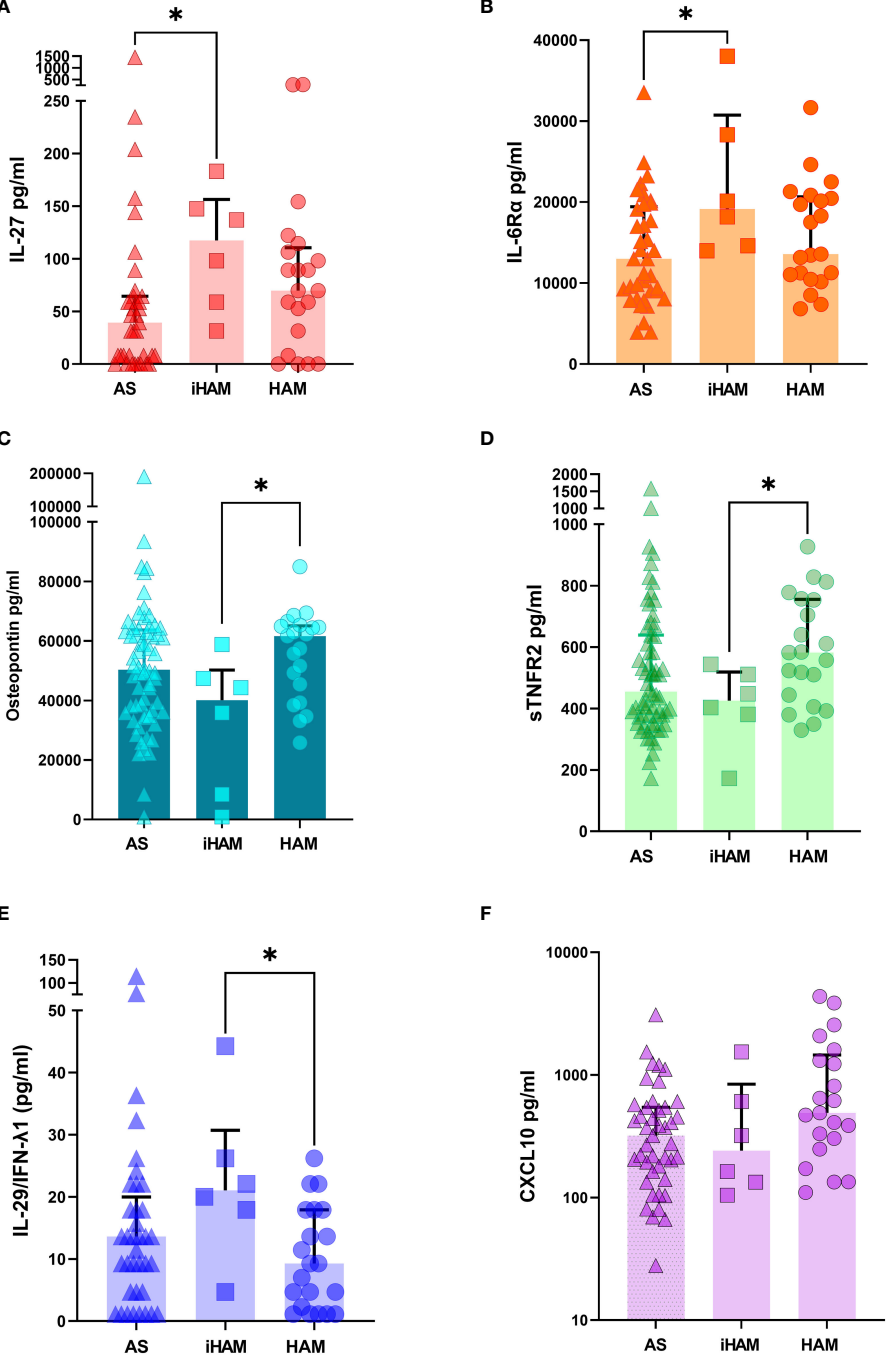
Additional candidate plasma biomarkers for incident neuroinflammatory disease. For plasma samples with sufficient volume, Luminex assay (37-plex and additional CXCL10) was performed (n=64, of which 37 AS, 6 iHAM and 21 HAM). **(A)** IL-27 **(B)** IL-6R-α **(C)** Osteopontin **(D)** sTNFR2 **(E)** IL-29/IFNλ1 **(F)** CXCL10. *p<0.05 Mann-Whitney test.

Because of the striking findings of low IL-10 and IL-4 as independent risk factors for iHAM, and the predictive power of IL-10 for posterior PVL dynamics, we further explored the possible upstream and downstream mechanisms by examining a large panel of 37 pro-and anti-inflammatory cytokines, chemokines and immune mediators. Of those 37, seven were previously suggested as candidate biomarkers and/or therapeutic targets (MMP-1/2/3, TNFR1/2, IL-22, Osteopontin) (16–24) in HAM, while most of them were not previously interrogated at the protein level in HAM patients but have been demonstrated in other neuroinflammatory or neurodegenerative disorders, such as CHI3L1 (also known as YKL-40) in HIV-Associated Neurocognitive Decline (HAND) (25). Since only 64 out of 110 plasma samples were available (of which 6/9 iHAM) for these additional assays, we considered this an exploratory analysis because of the lower sample size. As shown in **Figure 3**, pro-inflammatory IL-27 (Mann-Whitney p=0.030) and soluble IL6 receptor alpha chain (sIL6Rα, Mann-Whitney p=0.031) were significantly increased in iHAM patients as compared to AS, further underlining that the IL-10 nadir in iHAM corresponds to an increased inflammatory state. In addition, we found three additional candidate biomarkers that discriminate between iHAM and HAM patients (**Figure 3**): osteopontin (Mann-Whitney p=0.022) and soluble TNF receptor 2 (sTNFR2, Mann-Whitney p=0.049) are significantly lower in iHAM patients, while IL-29, also known as IFN-λ1, was significantly increased in iHAM, as compared to HAM patients (Mann-Whitney p=0.019). Reiterating its role as a central player in iHAM, IL-10 was significantly correlated to Osteopontin (R=0.33, p=0.0075) and sTNFR2 (R=0.32, p=0.010), but not to any other cytokines or chemokines. In contrast, none of these additional candidate biomarkers were correlated to PVL.

## Discussion

HAM is the most common disease associated with HTLV-1 in Brazil, and *bona fide* biomarkers to predict PLwHTLV-1 at risk are directly needed. Several groups have suggested PVL as the major biomarker for clinical progression from AS to HAM, which has been observed in several cross-sectional studies (6, 26), but contradicted in a number of prospective cohort studies (12, 27), which might suggest a temporal component in the predictive capacity of PVL for incident HAM. This study’s result on systemic IL-10 as a predictive biomarker of both iHAM and PVL dynamics on clinical follow-up might reconcile these apparently conflicting findings.

Our group and others have shown that several proinflammatory cytokines and chemokines, such as IL-2, TNF, IFN-gamma, CXCL10 and MIP-1beta are significantly increased in HAM patients, as compared to AS, which has been extensively reviewed in (5, 28). In this study, we found that lower levels of systemic IL-10 at the time of entry into the cohort were significantly predictive of clinical progression to definite HAM compared to asymptomatic carriers whose clinical evolution remained unchanged.

This suggests that IL-10 may have a protective role against HAM development, which is in agreement with an increase in both the IFN-γ/IL-10 ratio and the frequency of persistent HTLV-1-infected clones demonstrated by Espíndola et al. (2015) in HAM patients, as compared to AS (29). Furthermore, IL-10-mediated signals, such as the activation of STAT3 and IRF4 pathways, have been implicated in increased proliferation in HTLV-1-infected T-cell clones derived from HAM patients. In an *in vitro* comparison of Brazilian AS and HAM patients, the addition of IL-10 or TGF-β had varying effects on IFN-γ production. Additionally, individuals who developed HAM showed a decrease in FOXP3 expression and reduced IL-10 and TGF-β cytokine production, which are known to suppress the immune response. Although IL-10 is generally known for its anti-inflammatory properties, it may also have proinflammatory functions, especially in the presence of type I IFN. We previously demonstrated a weak correlation between age and IL-10 levels, with the IL6/IL10 ratio significantly increased with age. This ratio may be linked to a higher mortality rate in HTLV-1-infected individuals (12).

Although our findings provide insight into the plausible role of IL-10 in the pathogenesis of HTLV-1-triggered neuroinflammation, IL-10 is currently not a potential therapeutic target, due to the absence of human clinical trials. Future research should focus on elucidating the underlying molecular and cellular mechanisms and possibly explore targeted therapeutic interventions upstream or downstream of IL-10 and/or IL-4. Following up on the initial finding of low IL-10 and IL-4 as independent risk factors for iHAM, we further explored a previously proposed biomarker (CXCL10), as well as a large panel of 37 pro-and anti-inflammatory cytokines, chemokines and immune mediators, of which 30 had not been studied at the protein level before in HAM patients.

Within this comprehensive panel, seven proteins had previously emerged as potential candidate biomarkers or therapeutic targets in the setting of HAM, including matrix metalloproteinases (MMPs) MMP-1, MMP-2, and MMP-3, tumor necrosis factor receptors (TNFRs) TNFR1 and TNFR2, interleukin-22 (IL-22), and Osteopontin (16–24). Most of the other 30 candidate protein biomarkers had been described in various other neuroinflammatory or neurodegenerative afflictions, e.g. CHI3L1, also known as YKL-40, in the context of HIV-Associated Neurocognitive Disorders (HAND) (25). Although exploratory in nature, due to the inherent limitations associated with a reduced sample size, our findings underscored the heightened inflammatory milieu characterizing iHAM. Notably, pro-inflammatory interleukin-27 (IL-27) and soluble interleukin-6 receptor alpha chain (sIL6Rα) were significantly increased in iHAM patients relative to asymptomatic carriers (AS). IL-27 was significantly correlated to the CD4/C8 ratio (p=0.0099), but not to IL-10 levels, suggesting different cellular pathways at play with each cytokine. Both IL-27 and sIL6RA were not significantly correlated to sex, age or proviral load (data not shown).

Furthermore, our exploration yielded three novel candidate biomarkers for distinguishing iHAM from HAM patients: Osteopontin, soluble TNF receptor 2 (sTNFR2) and IL-29, also known as IFN-λ1. Of interest, another member of the IFN-lambda family, IFN λ3, was proposed as a possible genetic biomarker for HAM in both Brazilian and Spanish cohorts (30–33). Blocking TNF signaling through its receptors TNFR1 and TNFR2 has been widely targeted in clinical trials of autoimmune and inflammatory diseases, such as rheumatoid arthritis, Crohn’s disease, ankylosing spondylitis, psoriasis, and psoriatic arthritis. Thus, commonly used TNF inhibitors such as Infliximab, Etanercept, Adalimumab, Certolizumab pegol or Golimumab might be repurposed for the treatment of iHAM and HAM patients, in agreement with our previous findings of TNF as a negative predictor of corticosteroid response in HAM patients (13).

Several strengths and limitations of the current study are noteworthy. First, this prospective cohort study is, to our knowledge, the largest (clinical follow-up of 615 AS) and longest (total follow-up 6,673 person-years, ranging from 2–21 years) to date to report on incident HAM and the identification of non-invasive (plasma) biomarkers. Nevertheless, only 11 iHAM cases were identified in this study, of which 9 had available plasma samples, which should be considered a limitation of the study. An inherent limitation due to the neglected situation of PLwHTLV-1 in Brazil is the unknown time to onset of iHAM, since both the timing of infection as well as the time of diagnosis of definite HAM are expected to be delayed under the public health conditions during the study period. Practically, time of entry in the cohort upon HTLV-1 diagnosis is a poor proxy for time of infection, while the diagnosis of definite HAM requires inclusion of CSF analysis according to Castro-Costa criteria, causing a delay in iHAM classification. Logistic and financial limitations also made that frozen cells were not collected at entry into the cohort, which precluded elucidation of the IL-4 and IL-10 producing immune cell types. Likewise, no comparisons could be made to other proposed HAM biomarkers, such as Hbz mRNA (34) or CD4/CD8 activation markers (35) measured by flow cytometry. However, due to the logistic advantages of plasma storage (-80 or -20°C) and cytokine analysis, the candidate biomarkers proposed in this study are a more feasible option for large prospective cohort analysis, especially in resource-limited settings, as compared to storage of frozen cells (requiring liquid nitrogen storage) and multiparameter flow cytometry.

In summary, plasma anti-inflammatory cytokines IL-10 and IL-4 are non-invasive independent predictors of incident HAM cases in a large Brazilian cohort. While these major cytokines might not represent direct therapeutic targets, we also identified IL-29/IFN-ƛ1 and soluble receptors IL6R alpha (targeted by Tocilizumab) and TNFR2 (targeted by several biologics), which have been safely and effectively applied in several clinical trials of other inflammatory diseases, representing untapped potential for future clinical trials in HAM.

## Data availability statement

The original contributions presented in the study are included in the article/**Supplementary Material**. Further inquiries can be directed to the corresponding author.

## Ethics statement

The studies involving humans were approved by Instituto de Infectologia Emilio Ribas”, Sao Paulo-Brazil, approved the protocol (Number 07688818.2.1001.0061). The studies were conducted in accordance with the local legislation and institutional requirements. The participants provided their written informed consent to participate in this study.

## Author contributions

TA: Conceptualization, Investigation, Methodology, Project administration, Writing – original draft, Writing – review & editing. SM: Investigation, Methodology, Validation, Writing – review & editing. FG: Investigation, Methodology, Supervision, Validation, Writing – review & editing. VF: Formal Analysis, Validation, Writing – review & editing. MB: Data curation, Investigation, Writing – review & editing. JS: Data curation, Investigation, Supervision, Writing – review & editing. MH: Data curation, Investigation, Supervision, Writing – review & editing. RM: Data curation, Formal Analysis, Project administration, Writing – review & editing. FD: Investigation, Validation, Writing – review & editing. AO: Conceptualization, Supervision, Validation, Visualization, Writing – review & editing. EV: Methodology, Resources, Writing – review & editing. SC: Methodology, Resources, Writing – review & editing. DD: Funding acquisition, Supervision, Writing – review & editing. JV: Formal Analysis, Validation, Writing – review & editing. DS: Methodology, Resources, Writing – review & editing. JC: Investigation, Writing – original draft, Writing – review & editing. JVW: Formal Analysis, Investigation, Methodology, Supervision, Validation, Writing – original draft, Writing – review & editing.
