# Supplementary material for: IL-10 predicts incident neuroinflammatory disease and proviral load dynamics in a large Brazilian cohort of people living with human T-lymphotropic virus type 1
Source: Front Immunol. 2024 Jun 19;15:1416476. doi: 10.3389/fimmu.2024.1416476 (PMC11219816; doi:10.3389/fimmu.2024.1416476)
Supplement: Supplementary file 1 [file Table_1.docx]

Supplemental Materials

Table S1. Demographics, viral and immunological data

| **Variable** | **AS**  **59 (53.64%)** | **iHAM**  **09 (8.18%)** | **HAM/TSP**  **42 (38.18%)** |
| --- | --- | --- | --- |
| **Age (Years)**  Mean (±SD)  Median (25%-75% Percentile) | 51.24  (±13.10)  52.20  (41.8 - 59.6) | 51.59  (±15.85)  53.20  (35.2 - 65.35) | 49.22  (±13.50)  52.3  (41.05 - 57.6) |
| **Gender** |  |  |  |
| Male | 20 (33.89%) | - | 14 (33.34%) |
| Female | 39 (66.11%) | 09 (100%) | 28 (66.66%) |
| **Log HTLV-1 DNA Proviral Load**  Mean (±SD)  Median (25%-75% Percentile) | 3.21  (±1.49)  3.538  (2.561 - 4.470) | 3.86  (±1.61)  4.228  (3.163 - 4.801) | 4.17  (±1.41)  4.686  (3.243 - 5.701) |
| **Log HTLV-1 DNA Proviral Load follow-up**  Mean (±SD)  Median (25%-75% Percentile) | 3.57  (±1.17)  3.716  (2.819 - 4.574) | 4.61  (±0.66)  4.228  (4.096 - 5.302) | 4.51  (±1.03)  4.744  (4.376 -5.138) |
| **Spontaneous Lymphoproliferation (CPM*)**  Mean (±SD)  Median (25%-75% Percentile) | 2573  (±2588)  1776  (753 - 3720) | 2225  (±1423)  1839  (1174 - 3713) | 6265  (±7599)  3344  (1320 - 9114) |
| **CD4+ (cells/mm3)**  Mean (±SD)  Median (25%-75% Percentile) | 989.8  (±436.7)  1115  (596 - 1321) | 1455  (±194.5)  1479  (1261 - 1624) | 1181  (±470.1)  1105  (833 - 1451) |
| **CD8+ (cells/mm3)**  Mean (±SD)  Median (25%-75% Percentile) | 576.0  (±253.1)  477  (366 - 835.5) | 706.8  (±179.8)  678  (556.3 - 886) | 662.7  (±424.7)  462  (317 - 1001) |
| **CD4/CD8 ratio**  Mean (±SD)  Median (25%-75% Percentile) | 1.858  (±0.9422)  1.250  (1.165 - 2.855) | 2.163  (±0.6373)  2.090  (1.593 - 2.805) | 2.305  (±1.166)  2.095  (1.361 - 3.355) |

Note: *CPM = counts per minute
